# Effects of Visual Deprivation on Gait Dynamic Stability

**DOI:** 10.1100/2012/974560

**Published:** 2012-05-03

**Authors:** Marco Iosa, Augusto Fusco, Giovanni Morone, Stefano Paolucci

**Affiliations:** Clinical Laboratory of Experimental Neurorehabilitation, Fondazione Santa Lucia IRCCS, 00179 Rome, Italy

## Abstract

Vision can improve bipedal upright stability during standing and affect spatiotemporal parameters during walking. However, little is known about the effects of visual deprivation on gait dynamic stability. We have tested 28 subjects during walking under two different visual conditions, full vision (FV) and no vision (NV), measuring their upper body accelerations. Lower accelerations were found in NV for the reduced walking speed. However, the normalized accelerations were higher in the NV than in the FV condition, both in anteroposterior (1.05 ± 0.21 versus 0.88 ± 0.16, *P* = 0.001) and laterolateral (0.99 ± 0.26 versus 0.78 ± 0.19, *P* < 0.001) directions. Vision also affected the gait anteroposterior harmony (*P* = 0.026) and, interacting with the environment, also the latero-lateral one (*P* = 0.017). Directly (as main factor of the ANOVA) or indirectly (by means of significant interactions with other factors), vision affected all the measured parameters. In conclusion, participants showed an environment-dependent reduction of upper body stability and harmony when deprived by visual feedback.

## 1. Introduction

Maintaining balance during walking is one of the fundamental motor skills needed in bipedal locomotion. This dynamic stability can be defined as the capacity to move the body segments in a coordinated fashion so that the body can be displaced with a proper speed, keeping it more constant as possible for the conservation of momentum [1, 2] and minimizing upper body oscillations and hence the risk of fall [3, 4]. In fact, in an unstable gait, walking speed fluctuates, causing higher accelerations and hence inertial forces and perturbations that need to be controlled. Conversely, stable walking suitably exploits information about the orientation of the swaying body in respect to the environment mainly provided by vestibular system, lower extremities mechanoreceptors, and vision [5, 6]. The visual system can provide information not only about target distance and presence of obstacles, but also in maintaining balance during walking [7] and in adjusting trajectories when an obstacle appears or if the target is shifted [8].

Already in 1946, Edwards observed a small but significant increase in subjects' sway when standing under low illumination [9]. Many other successive studies investigated the impact of vision on standing balance. Some other ones investigated the effects on spatio-temporal gait parameters and joint kinematics of alterations in the optic flow field [10] or of visual deprivation [11–13]. However, little is known about the effects of visual deprivation on gait dynamic stability.

Hallemans and colleagues have recently performed an interesting cohort of studies about the effects of visual deprivation on the biomechanics of gait patterns [7, 11–13]. One of these studies was focused on the gait dynamic stability [7]. In this study, the authors did not found any difference in terms of trunk and pelvis angular range of motion between full- and no-vision conditions in healthy subjects. Conversely they found interesting results comparing healthy adults with those with visual impairments. The absence of upper body differences between the two visual conditions for healthy subjects could be imputable to many different reasons: the range of motions were not normalized for the different walking speeds, the gait analysis was carried out in an uncluttered environment (the experimental setting, as the authors properly highlighted), upper body accelerations were not investigated, and possible gender differences were not taken into account (especially because the main statistical analysis was not pairwise within subjects and between conditions).

In another study, in fact, trunk accelerations and interstep trunk-acceleration variability increased when light was suddenly reduced [14]. In this last study, the assessment of the ability to maintain balance during walking was performed using accelerometers, in accordance with the most recent literature [1, 4, 15]. Furthermore, the accelerometric parameters were compared between the two visual conditions for similar walking speeds. However, this study investigated the modulation of gait during the sudden transition from normal to marginal lighting, and not blind walking.

Furthermore, there are other two important factors to take into account for assessing the effects of visual deprivation on gait dynamic stability: gender and environment. Gender differences have already been observed in terms of upper body accelerations during walking [16]. Furthermore, locomotor control was found different between indoor and outdoor environments during blindfolded [17] and normal [18] walking, between gaits on different surfaces [1], between overground versus treadmill walking [19], and between gaits under different conditions of optic flow [10].

The aim of the present study was to investigate subjects' upper body accelerations during walking without visual feedback. The relationships between changes in gait spatio-temporal parameters and gait stability were also investigated. Because of the above reasons, male and female participants were tested indoors and outdoors, and both gender and environment have been included in this study as factors under investigation.

## 2. Materials and Methods

### 2.1. Participants and Protocol

Twenty-eight healthy volunteers were enrolled in this study (mean age: 28.14 ± 5.04 years). They were asked to stand on a line marked on the floor and then to walk straight for 10 m at a self-selected speed until arriving at another line taped on the ground. To highlight the target, an experimenter stood on the arriving line and moved away immediately before subjects started to walk (as previously explained to the subjects). Firstly, they were tested in walking to the target blindfolded (no vision, NV), then under visual control (full vision, FV).

To take into account also the possible gender differences in upper body acceleration during walking [16] each subgroup was formed by 7 females and 7 males. To take into account the possible influence of environment [17], fifteen participants were tested in an indoor hall (IN) and an other fifteen participants in an outdoor paved area (OUT) in the middle of a big lawn. Both environments have a length of about 18 m and a width of about 4 m, and they were quiet and well illuminated.

Both environment and gender had been used as between-subject factors.

Our subjects were naïve to blind without any previous practice of blindfolded walking in the two environments before testing to avoid drift after-effect due to previous practice [20]. For this reason, two different groups of subjects were tested in the two environments instead of a test-retest study design because memory can play a fundamental role into blindfolded walking [20].

This study was conducted in accordance with the Declaration of Helsinki about experiments on human subjects, and it was approved by the local ethical committee. Signed informed consent was obtained from each participant. Finally, this study was part of the project Assessment of Altered Motor Schemas financed by the Italian Ministry of Health (Grant RC11G.15).

### 2.2. Measurement Settings

**Figure 1:**
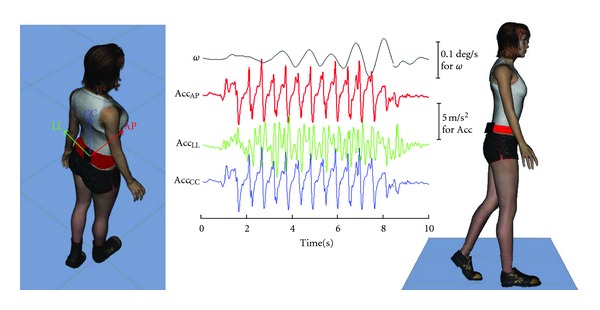
Schematic representation of gait stability assessment. A schematic representation of the walking test performed by a subject and the collected raw signals. Each subject performed the test wearing the red belt including the black device located on the back. Raw signals of cranio-caudal angular velocity (*ϖ*, black) and of acceleration signals along antero-posterior (Acc_AP_, red), latero-lateral (Acc_LL_, green), and cranio-caudal (Acc_CC_, blue) axes are shown.

During the test, subjects wore an elastic belt with a wearable inertial sensor device (FreeSense, Sensorize s.r.l., Rome; sampling frequency = 100 Hz) located on an area of their back corresponding to the L2-L3 spinous processes, close to their body center of mass, as schematically shown in Figure 1. This device is lightweight (93 g) and contains a triaxial accelerometer to measure accelerations along the three body axes (antero-posterior, AP; latero-lateral, LL; and craniocaudal, CC) and three gyroscopes to measure angular velocities around the above axes. During the test, all the subjects wore their commonly used shoes (avoiding special shoes such as high-heeled ones, flip-flops, ballerinas, or boots).

### 2.3. Parameter Computation

In the NV condition, participants were asked to stop walking when they thought they had achieved the target and to maintain that position. An experimenter measured their walked distance (WD) with a graduated tape. Time and the number of performed steps (Time and Ns) were determined from the recorded peaks of AP acceleration (see Figure 1); the mean step frequency was computed as SF = Ns/Time and the mean walking speed as WS = WD/Time [17].

Upper body accelerations were analyzed after the subtraction of their mean values and after low-pass filtering at 20 Hz and were summarized in six parameters for each body axis [4, 15, 16]. The parameters related to gait stability were averaged among the three values of three consecutive steps performed in the central part of the walking pathway and included the following: the acceleration root mean square (aRMS), a measure of acceleration dispersion, which coincides with the standard deviation because of signal mean subtraction [1]; the acceleration harmonic ratio (aHR), the ratio between the sum of even/odd (for AP and V) or odd/even (for LL) harmonic amplitudes calculated via the discrete Fourier transform, an indicator of the smoothness and rhythmicity of acceleration patterns [1]. To take into account the expected slower gait in NV, we have also computed the accelerations normalized in respect to walking speed [21]: nRMS = aRMS·SL/WS^2^, and the ratio between AP and LL components in respect to CC components analyzing aRMS_AP_/aRMS_CC_ and aRMS_LL_/aRMS_CC_ [22]. 

To take into account unsteady movements (beginning of walking and its stop), aRMS was computed also on the entire walking trial (atRMS). Furthermore, the mean angular velocity around cranio-caudal axis (*ϖ*) and its absolute value (|*ϖ*|) were computed to assess the mean rotation in the horizontal plane during the entire trial.

### 2.4. Statistical Analysis

The mean ± standard deviation was computed for all the investigated parameters. Repeated measure analysis of variance was performed on the above computed parameters along each direction to take into account the roles of the following factors: vision (V: FV versus NV, with-in subject factor), environment (E: IN versus OUT, between subject factor), gender (G: male versus female, between subject factor), and all their possible interactions.

Linear regression was used to assess the relationship between parameters, such as between aRMS and WS. The relevant Pearson coefficient (*R*) was used to assess the strength of these correlations. SPSS 17.0 was used for all statistical analyses, and the significance level was set at 0.05.

## 3. Results

All the estimated gait spatio-temporal parameters resulted highly changed when vision was excluded. When blindfolded, participants walked for a shorter distance in a longer time. So, their walking speed resulted reduced of about 26% due to a reduction of step length of about 16% and of step frequency of about 11% (see Table 1).

The reduction of walking speed in the NV condition implied the reduction of upper body accelerations (with similar results for aRMS and atRMS, see Table 2). However, the values of accelerations normalized for velocity, resulted significantly higher in the NV condition, especially along latero-lateral (27%) and antero-posterior (19%) axes. Interestingly, this change in latero-lateral direction was mainly imputable to male subjects (FV: 0.78 ± 0.23, NV: 1.14 ± 0.25; +48%, *P* < 0.001, paired *t*-test), whereas for females this change was lower and not statistically significant (FV: 0.78 ± 0.14, NV: 0.84 ± 0.18, +7%, *P* = 0. 277).

The mean angular velocity, as well as its absolute value, did not result significantly affected by visual deprivation. It means that the final position of subjects was translated but not rotated in respect to their starting position. However, we have observed in some subjects the presence of a lateral translation during blindfolded walking, but we did not measure these lateral errors. Also the normalized cranio-caudal acceleration was not affected by vision, confirming that cranio-caudal accelerations is usually less informative than antero-posterior and latero-lateral ones [1, 22].

Vision affected the harmony of gait only in antero-posterior direction. In fact, aHR_AP_ resulted significantly lower during blindfolded walking (NV) than during walking under full-vision condition (FV). On the other hand, aHRLL resulted affected by the interaction between visual and environmental factor: aHRLL resulted higher indoors than outdoors when subjects walked in the FV condition (in: 4.40 ± 3.18 versus out: 2.54 ± 0.89), whereas the opposite when subjects walked in the NV condition (in: 2.97 ± 1.27 versus out: 3.43 ± 1.19).

**Figure 2:**
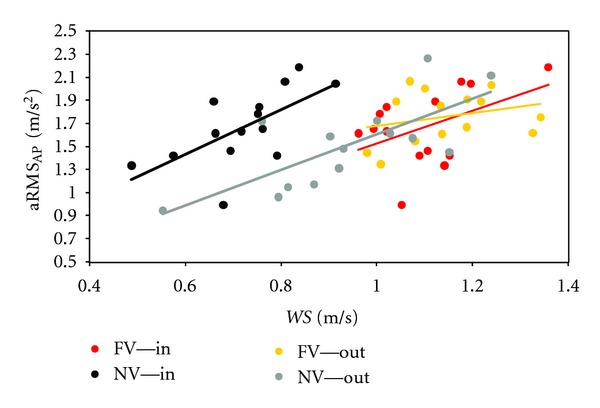
Effects of vision and environment on the relation between gait stability and speed. Values of antero-posterior acceleration root mean square are shown for all subjects in respect to their walking speed (together with the relevant regression lines) in the two visual conditions (FV versus NV) and the two environments (in versus out).

**Figure 3:**
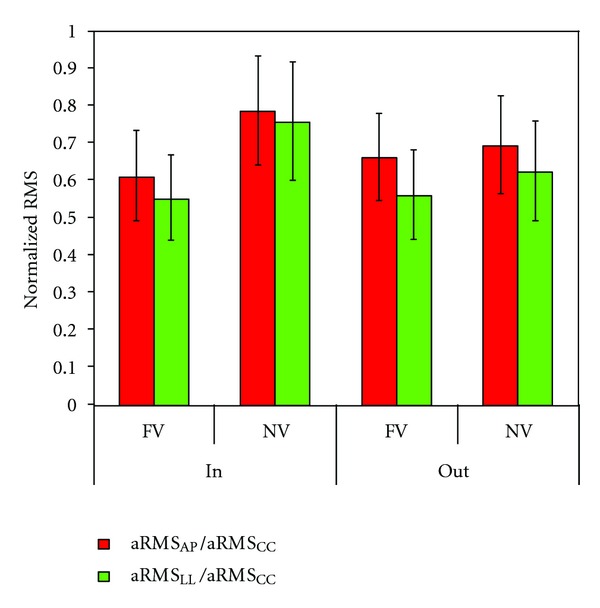
Effects of visual deprivation on gait stability in two different environments. Mean ± standard deviation of the normalized values of acceleration root mean square along antero-posterior (red bars) and latero-lateral (green bars) directions in full-versus. no-vision conditions (FV versus NV) and in the two environments (in versus out).

This interaction between vision and environment greatly affected also other gait parameters, both spatio-temporal ones and those related to dynamic stability.  Figure 2 shows the aRMS_AP_ values in respect to the relevant WS in the two visual conditions and in the two environments. The relationship between aRMS_AP_ and WS was similar between all subjects in the FV condition. In both the environments, the WS was reduced in the NV condition. However, outdoors, this WS reduction did not affect the linear relationship with the upper body accelerations and the regression line was a sort of continuation of the observed relationship in the FV condition. Conversely, indoors, the relationship between aRMS_AP_ and WS was still linear, but shifted in the range of higher accelerations. Finally, aRMS_AP_/aRMS_CC_ and aRMS_LL_/aRMS_CC_ resulted more affected by visual deprivation indoors than outdoors (Figure 3).

## 4. Discussion

Our results showed that visual deprivations affected gait dynamic stability. Previous studies have already showed that visual deprivation in healthy subjects affected their spatio-temporal gait parameters [11], lower limb kinematics [12], interlimbs coordination [13], and trunk stability during visual adaptation to dark [14]. Our study highlighted that, during target-directed walking, visual deprivation affected gait dynamic stability in a different manner between two different environments.

 In accordance with previous studies, we have observed a slower preferred walking in the NV condition [7, 11]. This can be a conservative strategy allowing for more time for haptic foot exploration and reducing uncertainty and fear (of falling or hitting a wall) [7, 17].

In fact, our results showed that the reduced walking speed can limit the upper body accelerations, facilitating the dynamic balance control. The strategy of reducing walking speed for reducing upper body instabilities has been already observed in patients with stroke when they were overstrained by a prolonged walking [4] and in elderly with visual impairments [23].

In the full-vision condition, we have found values of upper body acceleration aRMS and aHR similar to those already found for healthy subjects in previous studies [1, 21, 24]. Also the observed gender differences were similar to those previously observed in latero-lateral direction, with higher accelerations for males than for females [16]. Interestingly, also the interaction between vision and gender affected upper body stability, confirming that males are less able to control LL accelerations.

These results were similar between parameters estimated in the central part of walking pathway (aRMS)  and those evaluated on the entire pathway of the trial including the beginning and the end of movements (atRMS). On the other hand, starting to walk in the dark, such as when people wake up in the night for example to go to the bathroom, can be different to adaptation to dark from normal lighting, such as when people entered in a dark room coming from an illuminated one [14]. As expected, the normalized accelerations along both AP and LL axes resulted higher in the NV condition, indicating that the capacity to control dynamic stability was challenging in blind walking [7]. It confirms that vestibular and proprioceptive information cannot fully compensate for the loss of visual information to produce a normal gait pattern [12]. However, O'Connor and Kuo [25] found that the visual feedback information is primarily used to control balance in the latero-lateral direction while in the antero-posterior one gait stability can be passively obtained through the dynamics of walking, in accordance with the biomechanical model proposed by Bauby and Ko [26]. In fact, they observed higher step width RMS than step length RMS in presence of an optic flow perturbation [25]. Conversely, we found that also AP-normalized accelerations were higher in the NV versus the FV condition. Many reasons are possible to explain this difference. First of all, despite the connection between step width and trunk kinematics [27], the upper body accelerations along AP and LL axes were found more related to the body dynamic stability than to the step width [28]. Moreover, the maintenance of balance can probably be more challenging also along AP direction when vision is completely excluded than when it is only perturbed. However, as suggested by O'Connor and Kuo [25], also our results depicted a scenario confirming that the processes involved in controlling movements along the AP and LL axes can be quite different. In fact, we found that aHR_LL_ was affected only by the interaction between vision and environment, strengthening that the movements in the LL direction could be less controlled, allowing for adaptation to different environmental contexts [25]. On the contrary, vision was the only factor affecting the aHR_AP_, that is, the harmony of gait along the main direction of straight walking.

Our results confirmed that vestibular, acoustic, and proprioceptive information cannot fully compensate for the visual deprivation to produce a normal gait pattern [12]. The vision-environment interaction affecting both gait spatio-temporal parameters and gait stability can be due to different but possibly coexistent reasons. The first one is that external acoustic feedbacks can differently affect the gait stability in the two different environments. In fact, the performances of subjects during blindfolded walking were found more similar in two different environments when also acoustic information were removed [17]. The second one is related to the potential role of the environment as a selective tuner between different strategies more based on sensory feedbacks or on internal representation of external world [17, 29]. Finally, also motor imagery can play a fundamental role during blindfolded walking. It is well known that the production of the basic locomotor rhythm is largely dependent upon activity of central pattern generators within the spinal cord [30]. However, real-life gait also depends upon supraspinal structures that are involved in adapting walking movements to environmental and motivational demands [31]. It is conceivable that walking in the dark requires an involvement of the cortical structures already shown to be involved in locomotion imagination, foot positioning, and dynamic postural control [32]. For example, cortical structures outside primary motor regions such as supplementary motor area and cerebellum were found related to subjects' estimation of the timing to cover a previously seen path, emphasizing their role in imagining gait movements [32]. Target-directed walking without visual support, more than just going for walk in the dark, can imply the need of imagining the relative movement of the target in respect of the subject [33].

Motor imagery commonly involves a blend of kinesthetic and visual forms of movement imagination [34]. Interestingly, it has been recently shown that kinesthetic motor imagery, more than visual motor imagery, can modulate body sway during balance control [35]. The environmental constraints can affect this kinesthetic motor imagery more than the visual one, with a higher involvement of cortical structures outside primary motor regions when higher gait stability is required [32]. Furthermore, the role of kinesthetic information seemed to be fundamental during gait development even in presence of visual feedback. In fact, toddlers showed the need of a foot kinesthetic exploration of a visible obstacle to walk over it [36]. On the other hand, some pathologies, such as Parkinson's disease, can alter the integration of kinesthetic information with other sensorial information altering the motor control [37].

Further studies are needed to investigate the potential role of motor imagery during walking without visual control, and probably about the capacity of walkers to imagine an optic flow for judging the remaining distance and/or time-to-contact with their target.

The accelerometric assessment of the capacity to control gait dynamic stability has been shown to be informative during aging [38] and in many different pathologies (such as stroke [4, 22], cerebral palsy [39], and dystrophy [40]). This capacity was found to be superior in people with a visual impairment [7]. Conversely, visual deprivation can highlight less severe dynamic instabilities, as observed for obese children [41], and as well-known by neurologists and physiotherapists administering the Functional Gait Assessment test [42].

The main limitations of this study was that more controlled tests were probably needed to deeply investigate the role of vision-environment interaction. Furthermore, we have neither taken into account lateral translations during blind walking nor step width. Conversely, in respect to previous researches, the advantage of our study was to have investigated the effects of visual deprivation on target-directed walking, a task more close to real-life situations than going for a walk in the dark. In conclusion, we found lower upper body accelerations during blindfolded walking due to the reduced walking speed. However, blindfolded subjects had higher accelerations than those expected for this self-selected slow walking, especially when indoors and if males. Also the gait harmony resulted reduced when visual feedback was excluded, and in a quite different manner between antero-posterior and latero-lateral direction. Finally, our results also suggest that visual feedback can allow for having the same gait patterns in different surroundings whereas visual deprivation can have environment-specific effects on gait dynamic stability.

## Figures and Tables

**Table 1:** Mean ± standard deviation of gait spatio-temporal parameters in full (FV) versus no vision (NV) condition, and the *P* values of the effects of vision (V), environment (E), gender (G), and all their possible interactions on step length (SL), step frequency (SF), walking speed (WS), walked distance (WD), and walked time (Time).

Parameters	FV	NV	V	E	G	V ∗ E	V ∗ G	E ∗ G	V ∗ E ∗ G
SL	0.64 ± 0.05	0.54 ± 0.09	**<0.001**	**0.022**	0.091	**0.009**	0.454	0.977	0.784
SF	0.87 ± 0.06	0.77 ± 0.10	**<0.001**	0.142	0.928	**0.011**	0.234	0.142	0.082
WS	1.12 ± 0.11	0.83 ± 0.18	**<0.001**	**0.002**	0.391	**0.013**	0.883	0.389	0.519
WD	10.00 ± 0.00	8.86 ± 1.57	**<0.001**	**0.005**	0.551	**0.005**	0.551	0.555	0.555
Time	8.98 ± 0.83	10.95 ± 2.11	**<0.001**	0.224	0.209	0.715	0.607	0.096	0.121

**Table 2:** Effects on gait stability parameters. Mean ± standard deviation of gait stability parameters in full- versus no-vision condition, and the *P*-values of the effects of vision (V), environment (E), gender (G), and all their possible interactions on upper body accelerations evaluated on three central strides (aRMS), over the entire trial (atRMS), acceleration root mean square normalized by walking speed (nRMS) and by cranio-caudal acceleration (*aRMS*_*LL*_ */* *aRMS*_*CC*_ and *aRMS*_*AP*_ */* *aRMS*_*CC*_), harmonic ratio (aHR), mean angular velocity (*ϖ*), and its module (|*ϖ*|).

Gait stability Parameters	FV	NV	V	E	G	V ∗ E	V ∗ G	E ∗ G	V ∗ E ∗ G
aRMS									
CC	2.76 ± 0.59	1.87 ± 0.68	**<0.001**	0.119	0.590	**<0.001**	0.283	0.982	0.683
LL	1.51 ± 0.36	1.23 ± 0.36	**<0.001**	0.251	0.714	**0.029**	**0.003**	0.799	0.731
AP	1.72 ± 0.28	1.32 ± 0.36	**<0.001**	**0.029**	0.467	**0.009**	0.127	0.839	0.796
atRMS									
CC	2.38 ± 0.46	1.64 ± 0.56	**<0.001**	**0.047**	0.558	**0.001**	0.404	0.953	0.868
LL	1.41 ± 0.26	1.17 ± 0.32	**<0.001**	0.092	0.257	**0.018**	**<0.001**	0.947	0.577
AP	1.55 ± 0.24	1.22 ± 0.32	**<0.001**	**0.010**	0.394	**0.004**	0.137	0.709	0.818
nRMS									
CC	1.41 ± 0.28	1.45 ± 0.31	0.451	0.934	0.275	**0.019**	0.089	0.471	0.664
LL	0.78 ± 0.19	0.99 ± 0.26	**<0.001**	0.306	**0.026**	0.558	**0.002**	0.693	0.574
AP	0.88 ± 0.16	1.05 ± 0.21	**0.001**	0.664	0.166	0.689	0.157	0.346	0.513
aRMS_LL_ */*aRMS_CC_	0.56 ± 0.12	0.69 ± 0.16	**<0.001**	0.147	0.242	**0.011**	**0.006**	0.882	0.793
aRMS_AP_/aRMS_CC_	0.64 ± 0.12	0.74 ± 0.14	**<0.001**	0.656	0.861	**0.004**	0.729	0.908	0.670
aHR									
CC	8.74 ± 3.34	8.10 ± 4.07	0.425	0.741	0.170	0.442	0.161	0.563	0.957
LL	3.47 ± 2.48	3.20 ± 1.23	0.554	0.230	0.751	**0.017**	0.400	0.725	0.876
AP	7.54 ± 2.95	5.95 ± 2.19	**0.026**	0.366	0.547	0.667	0.636	0.887	0.826
*ϖ*	1.71 ± 4.34	0.29 ± 2.70	0.085	0.143	0.877	0.691	0.135	0.384	0.568
|*ϖ*|	2.52 ± 3.91	1.59 ± 2.17	0.188	0.114	0.758	0.653	0.559	0.958	0.989
